# Neonatal deaths in infants born weighing ≥ 2500 g in low and middle-income countries

**DOI:** 10.1186/s12978-020-01013-7

**Published:** 2020-11-30

**Authors:** Sarah Saleem, Farnaz Naqvi, Elizabeth M. McClure, Kayla J. Nowak, Shiyam Sunder Tikmani, Ana L. Garces, Patricia L. Hibberd, Janet L. Moore, Tracy L. Nolen, Shivaprasad S. Goudar, Yogesh Kumar, Fabian Esamai, Irene Marete, Archana B. Patel, Elwyn Chomba, Musaku Mwenechanya, Carl L. Bose, Edward A. Liechty, Nancy F. Krebs, Richard J. Derman, Waldemar A. Carlo, Antoinette Tshefu, Marion Koso-Thomas, Sameen Siddiqi, Robert L. Goldenberg

**Affiliations:** 1 Aga Khan University, Karachi, Pakistan; 2 RTI International, Durham, NC USA; 3 Instituto de Nutrición de Centroamérica y Panamá, Guatemala City, Guatemala; 4 School of Public Health, Boston University, Boston, MA USA; 5 KLE Academy Higher Education and Research, J N Medical College, Belagavi, Karnataka India; 6 Moi University School of Medicine, Eldoret, Kenya; 7 Lata Medical Research Foundation, Nagpur, India; 8 University Teaching Hospital, Lusaka, Zambia; 9 University of North Carolina At Chapel Hill, Chapel Hill, NC USA; 10 Indiana School of Medicine, University of Indiana, Indianapolis, IN USA; 11 University of Colorado School of Medicine, Denver, CO USA; 12 Thomas Jefferson University, Philadelphia, PA USA; 13 University of Alabama At Birmingham, Birmingham, AL USA; 14 Kinshasa School of Public Health, Kinshasa, Democratic Republic of the Congo; 15 Eunice Kennedy Shriver National Institute of Child Health and Human Development, Bethesda, MD USA; 16 Department of Obstetrics and Gynecology, Columbia University School of Medicine, New York, NY USA

**Keywords:** Neonatal mortality, ≥ 2500 g neonatal mortality, Low and middle-income countries, Global network

## Abstract

**Background:**

Babies born weighing ≥ 2500 g account for more than 80% of the births in most resource-limited locations and for nearly 50% of the 28-day neonatal deaths. In contrast, in high-resource settings, 28-day neonatal mortality among this group represents only a small fraction of the neonatal deaths. Yet mortality risks for birth weight of ≥ 2500 g is limited. Knowledge regarding the factors associated with mortality in these babies will help in identifying interventions that can reduce mortality.

**Methods:**

The Global Network’s Maternal Newborn Health Registry (MNHR) is a prospective, population-based observational study that includes all pregnant women and their pregnancy outcomes in defined geographic communities that has been conducted in research sites in six low-middle income countries (India, Pakistan, Democratic Republic of Congo, Guatemala, Kenya and Zambia). Study staff enroll all pregnant women as early as possible during pregnancy and conduct follow-up visits to ascertain delivery and 28-day neonatal outcomes. We analyzed the neonatal mortality rates (NMR) and risk factors for deaths by 28 days among all live-born babies with a birthweight ≥ 2500 g from 2010 to 2018 across the Global Network sites.

**Results:**

Babies born in the Global Network sites from 2010 to 2018 with a birthweight ≥ 2500 g accounted for 84.8% of the births and 45.4% of the 28-day neonatal deaths. Among this group, the overall NMR was 13.1/1000 live births. The overall 28-day NMR for ongoing clusters was highest in Pakistan (29.7/1000 live births) and lowest in the Zambian/Kenyan sites (9.3/1000) for ≥ 2500 g infants. ≥ 2500 g NMRs declined for Zambia/Kenya and India. For Pakistan and Guatemala, the NMR remained almost unchanged over the period. The ≥ 2500 g risks related to maternal, delivery and newborn characteristics varied by site. Maternal factors that increased risk and were common for all sites included nulliparity, hypertensive disease, previous stillbirth, maternal death, obstructed labor, severe postpartum hemorrhage, and abnormal fetal presentation. Neonatal characteristics including resuscitation, hospitalization, congenital anomalies and male sex, as well as lower gestational ages and birthweights were also associated with increased mortality.

**Conclusions:**

Nearly half of neonatal deaths in the Global Network sites occurred in infants born weighing ≥ 2500 g. The NMR for those infants was 13.1 per 1000 live births, much higher than rates usually seen in high-income countries. The changes in NMR over time varied across the sites. Even among babies born ≥ 2500 g, lower gestational age and birthweight were largely associated with increased risk of mortality. Since many of these deaths should be preventable, attention to preventing mortality in these infants should have an important impact on overall NMR.

Trial registration: https://ClinicalTrials.gov Identifier: NCT01073475

## Background

The World Health Organization (WHO) defines a neonatal death as the death of a live born infant during the first 28 days of life. Of 5.7 million under-five deaths that occur annually, approximately 47% occur in the first 28 days [1]. Disparities in the 28-day neonatal mortality rate (NMR) exist across and within countries. The NMR is much higher in resource constrained countries than in well-resourced countries, although exceptions exist [2]. Babies with a birthweight ≥ 2500 g account for more than 80% of the births in most resource limited locations, and often account for nearly half of the neonatal deaths [3]. Because of the continuing focus on preterm and low-birthweight births, which are higher risk but account for only about half the neonatal deaths, information on mortality for babies born with a birth weight of ≥ 2500 g is limited. Knowledge regarding the trends and factors associated with mortality in these babies will help in identifying interventions that can result in lower mortality.

In many high-resource settings, substantial improvements have been made in neonatal outcomes, with deaths in babies with a birth weight of ≥ 2500 g substantially reduced [3]. For example, in 2015 in Europe, 94%–95% of births were ≥ 2500 g and these births accounted for 24% of the neonatal mortality [4]. In low-resource settings, the mortality among these infants is still substantially higher than observed in high-resource settings [2, 5]. Thus, while infants born < 2500 g have a higher risk of neonatal mortality compared to those ≥ 2500 g, because most of the births are ≥ 2500 g, these births potentially represent a large proportion of the potentially preventable deaths in low-resource settings [6].

The Maternal Newborn Health Registry (MNHR) of the Global Network for Women’s and Children’s Health Research (Global Network) is a pregnancy registry conducted in sites in low-resource countries in south Asia, sub-Saharan Africa and Central America [7, 8]. The MNHR data demonstrates high neonatal mortality rates across the participating surveillance sites of member countries [8–10]. Overall, the MNHR data show slowly improving but continuing high maternal and neonatal mortality rates over the years across all Global Network sites [10]. We sought to explore the trends and factors associated with mortality in babies born with a birth weight of ≥ 2500 g. These infants are generally term or late preterm and the overwhelming majority can survive with usual obstetric and newborn care.

## Methods

The Global Network’s MNHR is a prospective, population-based observational study that includes all pregnant women and their outcomes in defined geographic communities (clusters) [7, 8]. In these clusters there are approximately 300 to 500 births annually. There are currently 8–10 clusters at each of the sites in western Kenya, Zambia (Kafue and Chongwe), the Democratic Republic of the Congo (DRC) (North and South Ubangi Province), Pakistan (Thatta in Sindh Provence), India (Belagavi and Nagpur) and Guatemala (Chimaltenango). The MNHR was initiated at each of the study sites between 2008 and 2009, except for the DRC, which joined the Global Network in 2014.

Registry administrators (RAs) are generally paid community health workers or nurses who identify pregnant women in their respective areas and after consent, enroll them in the MNHR. Once a pregnant woman is identified, the RAs obtain basic health information at enrollment, record the date of last menstrual period or early ultrasound report to assess gestational age, obtain a hemoglobin assessment where possible, and record the height and weight of the pregnant woman. A follow-up visit is carried out following delivery to collect information on pregnancy outcomes as well as health care received during delivery. Information on the study outcomes is based on medical record reviews and birth attendant and family interviews. Birth weights for babies born in hospitals are available from the birth certificates or hospital records and for home deliveries, babies are weighed within 48 h of birth by the RAs using study scales. Where birth weights could not be obtained by scale, the weight was estimated to distinguish infants < 1500 g and < 2500 g. Only 0.4% of the birthweights in ≥ 2500 g infants were estimated. The timing of neonatal death is defined by the day of death. Because gestational age estimation is often difficult in these settings, the Global Network developed an algorithm to determine an estimate of preterm birth [7].

### Statistical analyses

For analyses, we combined sites in the same region with similar outcomes. For example, the NMRs for Zambia and Kenya were similar so they were combined. The DRC data were not combined with the other African sites both because this site had a substantially higher NMR and the DRC did not have data available for 2010–2013. Similarly, the NMRs for Nagpur and Belagavi, India were similar and were combined, while the Pakistani site NMR was substantially higher. The Guatemala site was the only one in Central America.

We conducted analyses to determine the risk factors associated with NMR, defined as any death that occurred from day 0 through 27, among live births ≥ 2500 g. To assess the relationship between these characteristics and ≥ 2500 g mortality, log-binomial models were used to obtain relative risk estimates for mortality modeled as a function of each characteristic independently. Each model included site and the interaction between site and the characteristic of interest in order to obtain site-specific relative risk estimates while controlling for the correlation of morality within clusters using generalized estimating equations.

Thus, this study analyzed NMR among babies born alive with a birth weight of ≥ 2500 g during the last 9 years (2010–2018) across Global Network surveillance sites and also describes the maternal, delivery and newborn characteristics associated with a higher risk of mortality in these infants.

### Ethical approvals

This study was reviewed and approved by all sites’ ethics review committees at INCAP, Guatemala; University of Zambia, Zambia; Moi University, Kenya; Aga Khan University, Pakistan; KLE University’s Jawaharlal Nehru Medical College, Belagavi, India; Lata Medical Research Foundation, Nagpur, India, and the Kinshasa School of Public Health, DRC. The institutional review boards at each U.S. partner university and the Data Coordinating Center (RTI International) also approved the protocol. All women provided informed consent for participation in the study, including data collection and the follow-up visits.

## Results

**Fig. 1:**
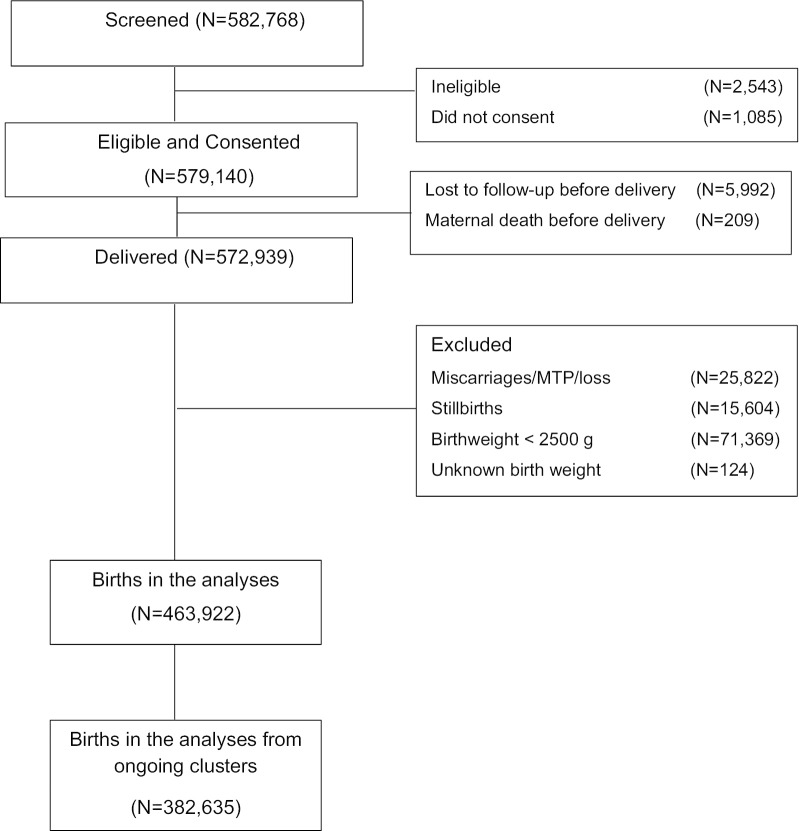
Subject diagram

From January 2010 through December 2018, 582,768 women were screened and 579,140 (99.4%) women consented to participate in the study (Fig. 1). Of those consented, delivery status was obtained for 572,939 women (98.9%) with 5992 lost to follow-up prior to delivery. There were 15,604 stillbirths, which were excluded from analyses. Altogether, 463,922 live births with a birth weight ≥ 2500 g were included in the study. Because several of the study clusters were discontinued over the course of the study period, and the DRC did not have data for all years, for analyses of trends over time, we restricted analyses to those clusters that were in the study for the entire period (N = 382,635).

Babies with a birth weight recorded as ≥ 2500 g accounted for 84.8% of the births in the Global Network sites and 45.4% of the neonatal deaths. The overall NMR for ≥ 2500 g infants for all sites from 2010 to 2018 was 13.1/1000 live births. Among clusters ongoing from 2010 to 2018, the overall NMR was 12.8 and declined from 15.0/1000 live births in 2010 to 10.8/1000 in 2018. (Table 1). For the babies who died, 48.6% died on days 0–1, 29.2% on days 2–6, 10.7% on days 7–13 and 11.5% on days 14–27.

**Table 1:** Neonatal mortality among infants ≥ 2500 g in the Global Network sites, 2010–2018

	Neonatal mortality rates overall and by year, n/N (rate/1000)					
	Total	Africa		South Asia		Central America
		Zambia/Kenya	DRC	India	Pakistan	Guatemala
2010–2018, All Clusters	6088/463,922 (13.1)	1193/128,580 (9.3)	325/27,300 (11.9)	1721/168,570 (10.2)	1941/70,123 (27.7)	908/69,349 (13.1)
2010–2018, Ongoing Clusters^a^	4884/382,635 (12.8)	1193/128,580 (9.3)	325/27,300 (11.9)	1269/130,297 (9.7)	1379/46,452 (29.7)	718/50,006 (14.4)
2010	670/44,605 (15.0)	205/15,302 (13.4)	–	217/18,394 (11.8)	172/6409 (26.8)	76/4500 (16.9)
2011	610/45,772 (13.3)	138/15,699 (8.8)	–	192/18,439 (10.4)	206/6583 (31.3)	74/5051 (14.7)
2012	550/44,032 (12.5)	134/14,702 (9.1)	–	191/18,370 (10.4)	165/5818 (28.4)	60/5142 (11.7)
2013	573/42,152 (13.6)	136/14,308 (9.5)	–	195/17,241 (11.3)	165/4963 (33.2)	77/5640 (13.7)
2014	539/42,301 (12.7)	114/13,608 (8.4)	76/5263 (14.4)	115/12,751 (9.0)	129/4876 (26.5)	105/5803 (18.1)
2015	498/42,431 (11.7)	121/13,981 (8.7)	56/5239 (10.7)	104/12,833 (8.1)	132/4398 (30.0)	85/5980 (14.2)
2016	534/41,912 (12.7)	142/13,882 (10.2)	57/5401 (10.6)	98/11,775 (8.3)	138/4421 (31.2)	99/6433 (15.4)
2017	492/40,820 (12.1)	112/13,616 (8.2)	76/5842 (13.0)	93/10,868 (8.6)	144/4525 (31.8)	67/5969 (11.2)
2018	418/38,610 (10.8)	91/13,482 (6.7)	60/5555 (10.8)	64/9626 (6.6)	128/4459 (28.7)	75/5488 (13.7)

^a^The following rows present ≥ 2500 g neonatal mortality in the subset of MNH Registry clusters collecting data during the entire period (i.e. 2010–2018 for all sites besides DRC and 2014–2018 for DRC) in order to evaluate trends

**Fig. 2:**
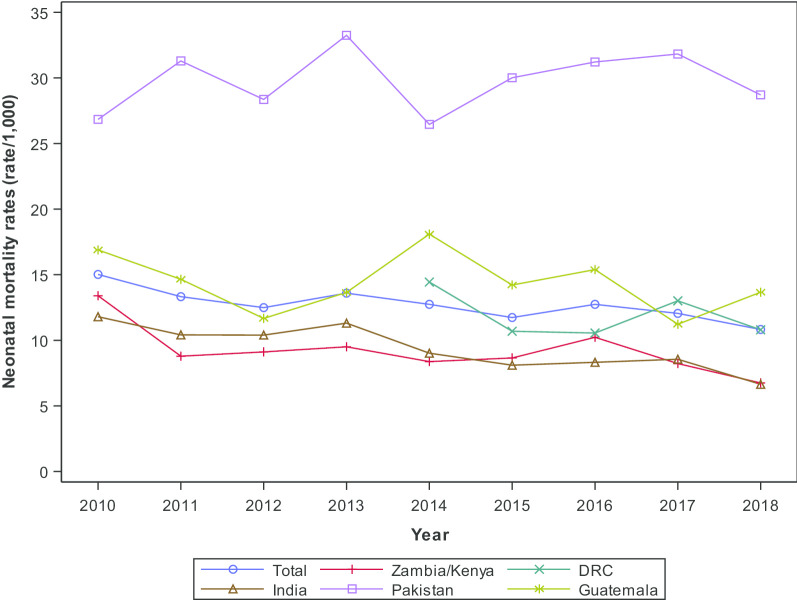
Neonatal death < 28-day trends in babies born alive and ≥ 2500 g by region and year, 2010–2018 ongoing clusters^1^. Data unavailable for DRC, 2010–2013.^1^ MNH Registry 2010–2018 deliveries in clusters collecting data during the entire period (i.e. 2010–2018 for all sites besides DRC and 2014–2018 for DRC)

Among ongoing clusters, the NMR for ≥ 2500 g infants was highest for the Pakistan site (29.7/1000 live births) and lowest for the Zambian/Kenyan sites (NMR 9.3/1000 live births) (Table 1). The NMR in babies born alive with a birth weight of ≥ 2500 g declined from 2010 to 2018 for the Zambia/Kenya sites from 13.4/1000 live births to 6.7/1000. The Indian sites had a decline in the ≥ 2500 g NMR from 11.8/1000 live births in 2010 to 6.6 /1000 live births in 2018. The ≥ 2500 g NMR in the Pakistani, DRC and Guatemalan sites did not appear to change substantially over time (Fig. 2).

Table 2 presents the numbers and percent of the maternal characteristics of the ≥ 2500 infants by who died and who lived in each site by region, and Table 3 describes the relative risk of maternal characteristics with neonatal death for infants born alive and with a ≥ 2500 g weight. In all sites, nulliparity was associated with a greater risk of ≥ 2500 g neonatal mortality as compared to women with 1–2 parity. The prior pregnancy ending in stillbirth was also associated with a higher risk of ≥ 2500 g neonatal mortality in all sites as was having a hypertensive disorder in pregnancy. In the DRC, although the numbers were small, ≥ 2500 g babies born to hypertensive mothers had a 17 times greater risk of dying, while at other sites the risk was more than twice compared to their counterparts. The risk for ≥ 2500 g neonatal mortality increased substantially when a maternal death occurred before 42 days post-partum in all study sites.

**Table 2:** Maternal characteristics for infants born alive and ≥ 2500, by Global Network region, 2010–2018

	Africa				South Asia				Central America	
	Zambia/Kenya		DRC		India		Pakistan		Guatemala	
	Newborn deaths by 28th day	Newborns survived by 28th day	Newborn deaths by 28th day	Newborns survived by 28th day	Newborn deaths by 28th day	Newborns survived by 28th day	Newborn deaths by 28th day	Newborns survived by 28th day	Newborn deaths by 28th day	Newborns survived by 28th day
Births, N	1193	127,387	325	26,975	1721	166,849	1941	68,182	908	68,441
Maternal age, N (%)	1187	126,754	325	26,949	1720	166,760	1939	68,041	907	68,429
< 20	338 (28.5)	28,755 (22.7)	76 (23.4)	4872 (18.1)	146 (8.5)	11,083 (6.6)	65 (3.4)	2613 (3.8)	126 (13.9)	11,141 (16.3)
20–35	766 (64.5)	90,462 (71.4)	206 (63.4)	19,817 (73.5)	1570 (91.3)	155,243 (93.1)	1743 (89.9)	61,725 (90.7)	621 (68.5)	50,296 (73.5)
> 35	83 (7.0)	7537 (5.9)	43 (13.2)	2,260 (8.4)	4 (0.2)	434 (0.3)	131 (6.8)	3703 (5.4)	160 (17.6)	6,992 (10.2)
Maternal Education, N (%)	1186	126,657	325	26,974	1712	166,267	939	68,011	908	68,432
No formal education	58 (4.9)	6,648 (5.2)	130 (40.0)	10,121 (37.5)	273 (15.9)	19,810 (11.9)	1,682 (86.7)	55,399 (81.5)	175 (19.3)	10,155 (14.8)
Primary/Secondary	1076 (90.7)	114,782 (90.6)	195 (60.0)	16,784 (62.2)	1265 (73.9)	124,862 (75.1)	226 (11.7)	10,698 (15.7)	715 (78.7)	55,002 (80.4)
University+	52 (4.4)	5,227 (4.1)	0 (0.0)	69 (0.3)	174 (10.2)	21,595 (13.0)	31 (1.6)	1914 (2.8)	18 (2.0)	3,275 (4.8)
Parity, N (%)	1188	126,797	325	26,974	1718	166,411	1894	66,675	908	68,436
0	440 (37.0)	35,763 (28.2)	82 (25.2)	4651 (17.2)	894 (52.0)	70,526 (42.4)	421 (22.2)	11,872 (17.8)	241 (26.5)	19,155 (28.0)
1–2	381 (32.1)	49,562 (39.1)	80 (24.6)	8574 (31.8)	708 (41.2)	87,428 (52.5)	533 (28.1)	22,750 (34.1)	262 (28.9)	26,770 (39.1)
≥ 3	367 (30.9)	41,472 (32.7)	163 (50.2)	13,749 (51.0)	116 (6.8)	8457 (5.1)	940 (49.6)	32,053 (48.1)	405 (44.6)	22,511 (32.9)
Number of ANC visits, N (%)	848	97,801	324	26,943	1138	117,633	1498	51,642	771	60,392
0	9 (1.1)	663 (0.7)	8 (2.5)	833 (3.1)	1 (0.1)	133 (0.1)	96 (6.4)	3,636 (7.0)	51 (6.6)	2,007 (3.3)
1	47 (5.5)	3845 (3.9)	10 (3.1)	1,192 (4.4)	16 (1.4)	2184 (1.9)	222 (14.8)	8141 (15.8)	38 (4.9)	2,706 (4.5)
2–3	396 (46.7)	46,420 (47.5)	137 (42.3)	11,794 (43.8)	304 (26.7)	26,452 (22.5)	613 (40.9)	22,142 (42.9)	212 (27.5)	15,036 (24.9)
≥ 4	396 (46.7)	46,873 (47.9)	169 (52.2)	13,124 (48.7)	817 (71.8)	88,864 (75.5)	567 (37.9)	17,723 (34.3)	470 (61.0)	40,643 (67.3)
Evidence of hypertensive disease/severe pre-eclampsia/eclampsia, N (%)	31 (2.6)	1,069 (0.8)	2 (0.6)	8 (0.0)	72 (4.2)	3,152 (1.9)	153 (7.9)	2,624 (3.9)	46 (5.1)	2,279 (3.3)
Last pregnancy was not a live birth^b^, n/N (%)	65/748 (8.7)	4634/91,028 (5.1)	16/243 (6.6)	736/22,323 (3.3)	42/824 (5.1)	2965/95,849 (3.1)	150/1473 (10.2)	3471/54,790 (6.3)	89/667 (13.3)	3,721/ 49,278 (7.6)
Maternal Death by 42 day follow-up, n/N (%)	8/1186 (0.7)	53/127,384 (0.0)	3/325 (0.9)	18/26,975 (0.1)	9/1721 (0.5)	88/166,849 (0.1)	7/1925 (0.4)	87/68,181 (0.1)	2/905 (0.2)	34/68,441 (0.0)

**Table 3:** Relative risk of neonatal death (< 28 day) by Global Network region and maternal characteristics for infants born alive and ≥ 2500 g, 2010–2018

	Adjusted RR for Neonatal Mortality for infants ≥ 2500 g (95% CI)^a^				
	Africa		South Asia		Central America
	Zambia/Kenya	DRC	India	Pakistan	Guatemala
Delivery Location					
Hospital	Ref	Ref	Ref	Ref	Ref
Clinic/Health Center	0.58 (0.38, 0.89)	0.43 (0.29, 0.64)	0.80 (0.71, 0.91)	0.95 (0.81, 1.11)	0.84 (0.51, 1.39)
Home/Other	0.66 (0.40, 1.09)	0.49 (0.32, 0.75)	1.35 (1.04, 1.76)	0.69 (0.61, 0.79)	1.07 (0.93, 1.24)
Delivery Mode					
Vaginal delivery	Ref	Ref	Ref	Ref	Ref
Assisted vacuum/forceps	4.50 (2.69, 7.53)	9.01 (3.15, 25.75)	4.82 (3.28, 7.07)	1.83 (1.53, 2.20)	2.38 (0.30, 19.11)
C-section	4.32 (3.36, 5.55)	6.98 (4.16, 11.70)	1.10 (0.98, 1.23)	1.32 (1.14, 1.52)	1.08 (0.95, 1.22)
Obstructed/prolonged labor/failure to progress	7.04 (5.48, 9.04)	15.49 (11.19, 21.44)	2.89 (2.23, 3.73)	2.71 (2.36, 3.10)	2.40 (2.09, 2.75)
Severe antepartum hemorrhage	4.80 (3.03, 7.61)	16.30 (8.70, 30.54)	4.23 (2.75, 6.52)	3.46 (2.89, 4.15)	1.90 (0.95, 3.79)
Severe postpartum hemorrhage	3.24 (2.41, 4.34)	6.94 (4.07, 11.84)	2.83 (1.78, 4.52)	2.05 (1.79, 2.34)	1.62 (1.14, 2.30)
Severe infection^b^	9.88 (6.13, 15.93)	12.07 (7.00, 20.80)	3.28 (1.32, 8.14)	2.99 (2.09, 4.28)	1.72 (0.91, 3.25)
Unplanned hospitalization^b^	8.56 (6.30, 11.63)	6.90 (4.24, 11.24)	2.85 (2.22, 3.66)	3.76 (2.00, 7.07)	2.84 (2.35, 3.44)
Abnormal fetal presentation	6.01 (4.69, 7.69)	8.12 (3.47, 19.01)	1.73 (1.30, 2.31)	2.74 (2.32, 3.23)	2.43 (1.72, 3.43)

^a^Relative risks (RR) and 95% confidence intervals from log-binomial models with generalized estimating equations to account for the correlation of outcomes within cluster. Neonatal mortality is modeled for each factor independently, accounting for site and the interaction of factor and site. Presented RR (95% CI) are for the interaction of factor and site to capture site-specific RRs

^b^Variable added to MNH forms in 2014

For Kenya/Zambia and DRC, mothers < 20 years of age were at higher risk for ≥ 2500 g neonatal mortality as compared to the mothers 20–35 year of age; however, this association was not observed for the sites in India, Pakistan, and Guatemala. Maternal age of more than 35 years was associated with a higher risk of ≥ 2500 g neonatal mortality in sites in the DRC, Pakistan, and Guatemala only. For the Indian, Pakistani, and Guatemalan sites, a lack of formal education was associated with increased ≥ 2500 g neonatal mortality, but not in the African region. Parity ≥ 3 was associated with an increased risk of ≥ 2500 g neonatal mortality in India, Pakistan, and Guatemala. The association between the number of antenatal care (ANC) visits and risk for ≥ 2500 g neonatal mortality varied by site. The risk of ≥ 2500 g neonatal mortality was higher in women who had a single ANC visit for Kenya/ Zambia but not for the other sites. As compared to women with ≥ 4 ANC visits, having no ANC doubled the risk of ≥ 2500 g neonatal mortality for women in the Guatemalan site, but was associated with reduced risk of neonatal death among Pakistani women. Two to three ANC visits were associated with higher risk for ≥ 2500 g neonatal mortality for the Indian sites but were associated with a 15% lower risk of neonatal death for the Pakistan site.

Table 4 presents the numbers and percent of the delivery characteristics of the ≥ 2500 g infants who died and lived in each site by region, and Table 5 describes the relative risk of delivery characteristics with neonatal death for infants born alive and with a ≥ 2500 g weight for each site. Abnormal fetal presentation at the time of delivery and obstructed labor were associated with higher risk of ≥ 2500 g neonatal mortality at all the sites. (Table 5) Severe post-partum hemorrhage and unplanned hospitalization were also associated with higher risk of ≥ 2500 g neonatal mortality for all the participating sites except Guatemala. Compared to hospital deliveries, babies with a birth weight ≥ 2500 g who were born at a clinic or health center had a lower risk of mortality in Zambia/Kenya, DRC and India; however, there was no difference in Pakistan and Guatemala. Home births were associated with higher risk of ≥ 2500 g NMR in the Indian sites, but associated with a 50% and 30% lower risk of NMR in the DRC and Pakistani sites, respectively. In the Guatemalan and Zambian/Kenyan sites, the risk of ≥ 2500 g neonatal mortality with home delivery compared to hospital delivery was similar. Except for Guatemala, assisted vacuum/forceps delivery was associated with a higher risk of ≥ 2500 g neonatal mortality. Similarly, cesarean delivery was associated with a higher risk of ≥ 2500 g neonatal mortality in Zambia/Kenya, DRC, and Pakistan, but not in India and Guatemala.

**Table 4:** Delivery characteristics for infants born alive and ≥ 2500, by Global Network Region, 2010–2018

	Africa				South Asia				Central America	
	Zambia/Kenya		DRC		India		Pakistan		Guatemala	
	Newborn deaths by 28th day	Newborns survived by 28th day	Newborn deaths by 28th day	Newborns survived by 28th day	Newborn deaths by 28th day	Newborns survived by 28th day	Newborn deaths by 28th day	Newborns survived by 28th day	Newborn deaths by 28th day	Newborns survived by 28th day
Births, N	1193	127,387	325	26,975	1721	166,849	1941	68,182	908	68,441
Delivery Location, N (%)	1193	127,386	325	26,975	1721	166,811	1939	68,163	908	68,441
Hospital	327 (27.4)	23,339 (18.3)	49 (15.1)	2185 (8.1)	1171 (68.0)	112,927 (67.7)	725 (37.4)	22,441 (32.9)	415 (45.7)	33,194 (48.5)
Clinic/Health Center	462 (38.7)	59,520 (46.7)	206 (63.4)	19,114 (70.9)	446 (25.9)	47,420 (28.4)	584 (30.1)	18,419 (27.0)	16 (1.8)	1,579 (2.3)
Home/Other	404 (33.9)	44,527 (35.0)	70 (21.5)	5676 (21.0)	104 (6.0)	6464 (3.9)	630 (32.5)	27,303 (40.1)	477 (52.5)	33,668 (49.2)
Delivery Mode, N (%)	1193	127,387	325	26,975	1721	166,849	1941	68,181	908	68,440
Vaginal Delivery	1091 (91.5)	124,806 (98.0)	301 (92.6)	26,705 (99.0)	1328 (77.2)	132,025 (79.1)	1558 (80.3)	57,997 (85.1)	688 (75.8)	52,118 (76.2)
Assisted vacuum/forceps	29 (2.4)	705 (0.6)	6 (1.8)	56 (0.2)	48 (2.8)	1007 (0.6)	112 (5.8)	2281 (3.3)	1 (0.1)	31 (0.0)
C-section	73 (6.1)	1876 (1.5)	18 (5.5)	214 (0.8)	345 (20.0)	33,817 (20.3)	271 (14.0)	903 (11.6)	219 (24.1)	16,291 (23.8)
Obstructed/prolonged labor/failure to progress, N (%)	317 (26.6)	6145 (4.8)	62 (19.1)	348 (1.3)	399 (23.2)	16,027 (9.6)	585 (30.2)	9146 (13.4)	106 (11.7)	3,599 (5.3)
Severe antepartum hemorrhage, N (%)	48 (4.0)	1029 (0.8)	6 (1.8)	26 (0.1)	17 (1.0)	390 (0.2)	135 (7.0)	1349 (2.0)	9 (1.0)	361 (0.5)
Severe postpartum hemorrhage, N (%)	88 (7.5)	2939 (2.3)	13 (4.0)	151 (0.6)	26 (1.5)	822 (0.5)	160 (8.2)	2807 (4.1)	23 (2.5)	1,110 (1.6)
Severe infection^a^, N (%)	34 (6.1)	380 (0.6)	23 (7.1)	152 (0.6)	4 (0.7)	137 (0.2)	52 (6.0)	602 (2.0)	8 (1.5)	354 (0.9)
Unplanned hospitalization^a^, N (%)	27 (5.6)	375 (0.7)	15 (5.4)	185 (0.8)	45 (9.5)	2,025 (3.6)	18 (2.4)	152 (0.6)	105 (20.6)	3,171 (8.2)
Abnormal fetal presentation, n/N (%)	67/1192 (5.6)	1198/127,328 (0.9)	9/325 (2.8)	89/26,955 (0.3)	47/1720 (2.7)	2797/166,584 (1.7)	142/1939 (7.3)	1829/68,119 (2.7)	56/908 (6.2)	1,803/ 68,418 (2.6)

**Table 5:** Relative risk of neonatal death (< 28 day) by Global Network region and delivery characteristics for infants born alive and ≥ 2500 g, 2010–2018

	Adjusted RR for Neonatal Mortality for infants ≥ 2500 g (95% CI)^a^				
	Africa		South Asia		Central America
	Zambia/Kenya	DRC	India	Pakistan	Guatemala
Delivery Location					
Hospital	Ref	Ref	Ref	Ref	Ref
Clinic/Health Center	0.58 (0.38, 0.89)	0.43 (0.29, 0.64)	0.80 (0.71, 0.91)	0.95 (0.81, 1.11)	0.84 (0.51, 1.39)
Home/Other	0.66 (0.40, 1.09)	0.49 (0.32, 0.75)	1.35 (1.04, 1.76)	0.69 (0.61, 0.79)	1.07 (0.93, 1.24)
Delivery Mode					
Vaginal delivery	Ref	Ref	Ref	Ref	Ref
Assisted vacuum/forceps	4.50 (2.69, 7.53)	9.01 (3.15, 25.75)	4.82 (3.28, 7.07)	1.83 (1.53, 2.20)	2.38 (0.30, 19.11)
C-section	4.32 (3.36, 5.55)	6.98 (4.16, 11.70)	1.10 (0.98, 1.23)	1.32 (1.14, 1.52)	1.08 (0.95, 1.22)
Obstructed/prolonged labor/failure to progress	7.04 (5.48, 9.04)	15.49 (11.19, 21.44)	2.89 (2.23, 3.73)	2.71 (2.36, 3.10)	2.40 (2.09, 2.75)
Severe antepartum hemorrhage	4.80 (3.03, 7.61)	16.30 (8.70, 30.54)	4.23 (2.75, 6.52)	3.46 (2.89, 4.15)	1.90 (0.95, 3.79)
Severe postpartum hemorrhage	3.24 (2.41, 4.34)	6.94 (4.07, 11.84)	2.83 (1.78, 4.52)	2.05 (1.79, 2.34)	1.62 (1.14, 2.30)
Severe infection^b^	9.88 (6.13, 15.93)	12.07 (7.00, 20.80)	3.28 (1.32, 8.14)	2.99 (2.09, 4.28)	1.72 (0.91, 3.25)
Unplanned hospitalization^b^	8.56 (6.30, 11.63)	6.90 (4.24, 11.24)	2.85 (2.22, 3.66)	3.76 (2.00, 7.07)	2.84 (2.35, 3.44)
Abnormal fetal presentation	6.01 (4.69, 7.69)	8.12 (3.47, 19.01)	1.73 (1.30, 2.31)	2.74 (2.32, 3.23)	2.43 (1.72, 3.43)

^a^Relative risks (RR) and 95% confidence intervals from log-binomial models with generalized estimating equations to account for the correlation of outcomes within cluster. Neonatal mortality is modeled for each factor independently, accounting for site and the interaction of factor and site. Presented RR (95% CI) are for the interaction of factor and site to capture site-specific RRs

Table 6 presents the numbers and percent of the newborn characteristics of the ≥ 2500 g infants who died and lived in each site by region, and Table 7 describes the relative risk of neonatal characteristics with neonatal death for infants born alive and with a ≥ 2500 g birthweight. Congenital anomalies were associated with adjusted relative risks of neonatal mortality in the various sites ranging from 9.7 to 101.0 and appeared to be the strongest risk factor for neonatal death in nearly all sites (Table 7). The next strongest risk factor for neonatal mortality among the infant characteristics was the receipt of resuscitation at birth with relative risks ranging from 6.6 in Pakistan to 48.2 in the DRC. Neonatal hospitalization was a significant risk factor in all sites with relative risks ranging from 10 in Zambia/Kenya to 66.4 in India. Male gender was also associated with increased risk of neonatal death in ≥ 2500 g births in all sites. The risk for ≥ 2500 g neonatal mortality was also significantly increased among those classified as preterm compared to term for all sites except the DRC. As compared to babies with a birth weight of 3001 to 3500 g, babies born with a birth weight of 2500 to 2700 g had a higher risk of death in Zambia/Kenya, India, and Pakistan. This higher risk of death continued for babies born with a birth weight of 2701 g to 3000 g for the Kenyan/Zambian and Pakistani sites. As compared to babies with a birth weight of 3001 to 3500 g, babies with a birth weight ≥ 3500 g were at higher risk of death in the sites in Zambia/Kenya, DRC, and Guatemala. Except for Pakistan, ≥ 2500 g neonatal mortality was higher in infants who were not placed on mother’s chest after delivery. Furthermore, in the African sites, the risk of ≥ 2500 g neonatal mortality was higher for multiple births compared to singletons.

**Table 6:** Newborn characteristics for infants born alive and ≥ 2500, by Global Network Region, 2010–2018

	Africa				South Asia				Central America	
	Zambia/Kenya		DRC		India		Pakistan		Guatemala	
	Newborn deaths by 28th day	Newborns survived by 28th day	Newborn deaths by 28th day	Newborns survived by 28th day	Newborn deaths by 28th day	Newborns survived by 28th day	Newborn deaths by 28th day	Newborns survived by 28th day	Newborn deaths by 28th day	Newborns survived by 28th day
Births, N	1193	127,387	325	26,975	1721	166,849	1941	68,182	908	68,441
Preterm, N (%)	930/1134 (82.0)	110,938/122,852 (90.3)	268/325 (82.5)	23,230/26,897 (86.4)	1565/1684 (92.9)	156,270/163,795 (95.4)	1312/1813 (72.4)	59,129/66,112 (89.4)	815/883 (92.3)	63,207/ 67,118 (94.2)
Birthweight, N (%)	1,041	126,930	305	26,949	1705	166,611	1,327	67,948	884	68,423
2500–2700 g	181 (17.4)	12,328 (9.7)	54 (17.7)	4630 (17.2)	760 (44.6)	65,213 (39.1)	568 (42.8)	16,851 (24.8)	101 (11.4)	8,388 (12.3)
2701–3000 g	357 (34.3)	39,143 (30.8)	104 (34.1)	9378 (34.8)	648 (38.0)	67,666 (40.6)	543 (40.9)	25,440 (37.4)	330 (37.3)	25,010 (36.6)
3001–3500 g	300 (28.8)	53,179 (41.9)	102 (33.4)	9888 (36.7)	260 (15.2)	29,921 (18.0)	166 (12.5)	19,863 (29.2)	309 (35.0)	27,511 (40.2)
> 3500 g	203 (19.5)	22,280 (17.6)	45 (14.8)	3053 (11.3)	37 (2.2)	3811 (2.3)	50 (3.8)	5794 (8.5)	144 (16.3)	7,514 (11.0)
Not placed on mother's chest after delivery, n/N (%)	525/1151 (45.6)	32,450/126,743 (25.6)	147/301 (48.8)	6261/26,962 (23.2)	1148/667 (68.9)	73,206/163,580 (44.8)	1798/1930 (93.2)	62,948/68,123 (92.4)	643/885 (72.7)	39,220/ 67,460 (58.1)
Multiple Birth, n/N (%)	47/1193 (3.9)	1436/127,383 (1.1)	11/325 (3.4)	271/26,975 (1.0)	7/1721 (0.4)	481/166,832 (0.3)	11/1936 (0.6)	415/68,154 (0.6)	5/908 (0.6)	204/68,441 (0.3)
Gender, N (%)	1193	127,373	325	26,974	1720	166,842	1941	68,175	908	68,438
Male	675 (56.6)	64,954 (51.0)	193 (59.4)	14,110 (52.3)	1023 (59.5)	87,787 (52.6)	1114 (57.4)	35,969 (52.8)	528 (58.1)	35,462 (51.8)
Female	518 (43.4)	62,419 (49.0)	132 (40.6)	12,864 (47.7)	697 (40.5)	79,055 (47.4)	827 (42.6)	32,206 (47.2)	380 (41.9)	32,976 (48.2)
Congenital anomaly, N (%)	11 (4.0)	140 (0.1)	1 (1.3)	6 (0.0)	63 (12.3)	172 (0.1)	41 (5.6)	397 (0.6)	27 (6.0)	175 (0.3)
Hospitalization, N (%)	56 (22.8)	3776 (3.2)	7 (10.6)	67 (0.3)	186 (37.8)	1295 (0.8)	244 (35.9)	366 (0.6)	183 (41.2)	1,656 (2.4)
Resuscitation with bag and mask, N (%)	252 (21.6)	1622 (1.3)	137 (42.4)	282 (1.0)	753 (45.0)	3960 (2.4)	779 (40.3)	5765 (8.5)	130 (14.6)	770 (1.1)

**Table 7:** Relative risk of neonatal death (< 28 day) by Global Network Region and newborn characteristics for infants born alive and ≥ 2500 g, 2010–2018

	Adjusted RR for neonatal mortality for infants ≥ 2500 g (95% CI)^a^				
	Africa		South Asia		Central America
	Zambia/Kenya	DRC	India	Pakistan	Guatemala
Preterm	2.02 (1.57, 2.59)	1.33 (0.99, 1.79)	1.62 (1.33, 1.98)	3.09 (2.84, 3.36)	1.35 (1.16, 1.56)
Birthweight					
2500–2700 g	2.55 (1.96, 3.33)	1.14 (0.84, 1.55)	1.43 (1.17, 1.75)	3.95 (3.48, 4.49)	1.06 (0.79, 1.43)
2701–3000 g	1.61 (1.36, 1.90)	1.08 (0.80, 1.46)	1.12 (0.94, 1.34)	2.53 (2.16, 2.96)	1.16 (0.98, 1.38)
3001–3500 g	Ref	Ref	Ref	Ref	Ref
> 3500 g	1.57 (1.29, 1.92)	1.50 (1.02, 2.21)	1.12 (0.75, 1.66)	1.05 (0.82, 1.33)	1.71 (1.41, 2.07)
Not placed on mother's chest after delivery	2.44 (1.93, 3.09)	3.43 (1.75, 6.72)	3.18 (2.54, 4.00)	1.18 (0.95, 1.47)	1.87 (1.62, 2.17)
Multiple Birth	3.51 (2.67, 4.60)	3.40 (2.00, 5.76)	1.45 (0.74, 2.83)	0.93 (0.51, 1.70)	1.85 (0.91, 3.80)
Gender					
Male	1.25 (1.06, 1.47)	1.34 (1.08, 1.67)	1.33 (1.19, 1.48)	1.20 (1.08, 1.34)	1.29 (1.18, 1.41)
Female	Ref	Ref	Ref	Ref	Ref
Congenital anomaly	36.51 (20.67, 64.46)	49.79 (6.71, 369.67)	101.00 (75.32, 135.43)	9.66 (7.12, 13.10)	21.93 (15.86, 30.32)
Hospitalization	10.01 (4.39, 22.82)	38.70 (20.80, 71.99)	66.43 (45.04, 97.97)	57.27 (46.58, 70.41)	25.96 (18.67, 36.12)
Resuscitation with bag and mask, N (%)	18.55 (14.38, 23.92)	48.24 (36.86, 63.14)	29.60 (22.37, 39.16)	6.57 (5.80, 7.45)	13.29 (10.87, 16.26)

^a^Relative risks (RR) and 95% confidence intervals from log-binomial models with generalized estimating equations to account for the correlation of outcomes within cluster. Neonatal mortality is modeled for each factor independently, accounting for site and the interaction of factor and site. Presented RR (95% CI) are for the interaction of factor and site to capture site-specific RRs

## Discussion

We examined neonatal mortality in seven sites in six countries over the last 9 years and explored the NMR and its risk factors in babies born alive with a birth weight of ≥ 2500 g. Across all sites, ≥ 2500 g babies accounted for 85% of the live-born babies and 45.4% of the neonatal deaths. The NMR for these babies averaged 13.1 per 1000 live births across the sites from 2010 to 2018.

The Pakistan site had the highest ≥ 2500 g NMR at 29.7 per 1000 livebirths and the Kenyan/Zambian sites the lowest at 9.3/1000. We found that the ≥ 2500 g neonatal mortality rates declined over the last nine-year period for the Indian and Kenyan/Zambian sites but not in Pakistan and Guatemala where no remarkable changes in the ≥ 2500 g NMRs were observed.

Maternal factors generally associated with increased risk of ≥ 2500 g neonatal mortality were older or younger ages, no formal education, nulliparity or a parity of ≥ 3, hypertensive disorders of pregnancy, obstructed labor, a history of previous stillbirths, and maternal death. However, other risks varied across the sites. Lack of adequate ANC visits or less care has been documented as a risk factor for poor pregnancy outcomes [11]. However, the number of ANC visits required to reduce risk remains in question. The WHO for many years supported a recommendation of at least four visits in resource-limited countries. However, these guidelines were revised recently and now WHO recommends at least eight ANC visits to achieve better fetal and maternal outcomes [11]. We also found a higher risk of ≥ 2500 g neonatal mortality associated with reduced ANC visits during pregnancy at some sites. Maternal co-morbidities such as those found in this study can adversely affect the pregnancy outcomes and many are not sufficiently addressed in low-resource settings. Severe postpartum hemorrhage, hypertensive disease and unplanned hospitalization were common risk factors for ≥ 2500 g neonatal mortality for all the participating sites. The literature suggests that in case of maternal illness or death, the chances of survival for a newborn is decreased. Babies whose mothers are severely ill may be deprived of care such as breastfeeding and skin to skin contact which may result in an increased risk of morbidity and mortality [12].

An estimated 10–15% of pregnancies will have complications and might need a cesarean section or some other kind of assistance to facilitate delivery. In our study, ≥ 2500 g NMR was higher among those with vacuum and forceps assisted deliveries as compared to vaginal delivery in most sites. If timely intervention in the form of a cesarean section is not carried out for mothers for certain complications, the risk of mortality increases for the fetus, mother and newborn [13, 14]. With emphasis on facility delivery and skilled attendance at birth, more women in low-resource countries are opting for delivery at a hospital, clinic or a health center. Our results show that as compared to a hospital delivery, the risk for ≥ 2500 g mortality was less in babies who were born in a clinic or a health center in Zambia/Kenya, DRC and India but not for Pakistan and Guatemala. We do not know the reasons for why women or their care- givers chose a particular location for delivery, but it seems likely that more women with pregnancy complications deliver in hospitals in Zambia/Kenya and India, but perhaps less so in Pakistan and Guatemala. Home delivery was associated with an increased risk of ≥ 2500 g NMR only in the Indian sites.

For all sites except DRC, the ≥ 2500 g NMR risk was significantly higher among babies born preterm. Risk of NMR was also higher for multiple births in the African sites, male gender, those with congenital anomalies and those who were hospitalized at any time before 28 days of life. In high-income countries, almost all babies who are born with a birthweight of ≥ 2500 g survive, and the causes of mortality are different from those observed in low-income countries. In a countrywide study from the United States, sudden unexpected death of infants was the most common cause of infant mortality among infants born full term [15]. In contrast, common causes of ≥ 2500 g neonatal mortality in LMICs are prematurity, asphyxia and sepsis [5, 6].

The reported proportion with congenital anomalies in our sites was lower than reported from high- income countries, likely due to the low rates of X-rays, ultrasound and autopsies in these regions. Our results also show a higher risk of ≥ 2500 g neonatal mortality among babies born with congenital abnormalities at all the sites. Many anomalies cannot be treated in low-resource settings and that factor may account for some of the mortality associated with anomalies.

Resuscitation at birth, most likely due to pre-delivery asphyxia, was strongly associated with mortality at all sites. Infant hospitalization was also associated with ≥ 2500 g neonatal mortality at all sites.

Perinatal outcomes in twin or higher order multiple births, especially in resource-poor countries, are generally compromised. Our results also show a higher risk of mortality in twin births even with a birth weight of ≥ 2500 g. Maternal risk factors such as death, blood transfusion, intensive care unit admission or hysterectomy are higher in twin pregnancies as compared to singleton pregnancies [16].

The risk of ≥ 2500 g neonatal mortality was high for babies who were not put on mothers’ chest after delivery. Whether this was causal or could be explained by the fact that sick newborns were not placed on the mother’s chest, since they were receiving medical care, is unknown. In general, skin-to-skin contact after birth helps to maintain temperature, helps initiate early breast-feeding and promotes infant and maternal bonding [17].

This study had a number of strengths and some weaknesses. Among the strengths are the large sample size, multiple sites, prospective data collection and standard data collection protocols used across the sites. Weaknesses include the inability of some sites to collect all the data required. For example, data on anemia were inconsistently collected across the sites and were not analyzed for their association with ≥ 2500 g neonatal mortality. Blood pressure measurements were also not made consistently across the sites and the lack of measurements, especially in the DRC may account for the very high relative risk for hypertension reported from that site. Reporting of congenital anomalies was generally based only on external observation and was likely not consistently determined across the sites. Also, a small number of the birthweights were estimated.

## Conclusions

In summary, babies who were born with a birth weight of ≥ 2500 g accounted for 45% of all neonatal deaths across the Global Network. The NMR for these babies was highest for the Pakistan site and was lowest for the Zambian/Kenyan sites. A declining NMR was observed for Zambia/Kenya and India sites. In the Pakistan and Guatemala sites, the rates remained unchanged over the last nine years. The mortality risks in infants born at ≥ 2500 g were related to maternal, delivery and newborn characteristics. The NMR for those infants was 13.1 per 1000 live births, significantly higher than rates seen in high-income countries. The NMR and decline in mortality were not consistent across the sites. Even among infants ≥ 2500 g, lower gestational age and birthweight were largely associated with increased NMR. Since many of the deaths in infants with birthweight ≥ 2500 g should be preventable, attention to preventing mortality in these infants should have an important impact on overall NMR.

## Data Availability

The study data will be available through the NICHD Data and Specimen Hub (DASH). Available at https://dash.nichd.nih.gov/.

## Abbreviations

MNHR: Maternal Newborn Health Registry
NMR: Neonatal mortality rates
WHO: World Health Organization
DRC: Democratic Republic of the Congo
RAs: Registry administrators
